# Supplementary material for: Meta-Analysis and Gene Set Enrichment Relative to ER Status Reveal Elevated Activity of MYC and E2F in the “Basal” Breast Cancer Subgroup
Source: PLoS One. 2009 Mar 9;4(3):e4710. doi: 10.1371/journal.pone.0004710 (PMC2650420; doi:10.1371/journal.pone.0004710)
Supplement: Dataset S1 — GSEA results from first screen. The file contains a zipped archive of the HTML results of the second GSEA screen. Reports for enriched or depleted datasets may be accessed from the “index.html” link within the my_analysis.GseaPreranked.1219975950625.rpt.zip ZIP archive. Enrichment plots are available for each of the 50 most enriched and depleted gene sets. (13.32 MB ZIP) [file pone.0004710.s001.zip › my_analysis.GseaPreranked.1219975950625.rpt.zip!//AGUIRRE_PANCREAS_CHR17.html]

Details for gene set AGUIRRE\_PANCREAS\_CHR17[GSEA]

|  || Dataset | zSco.ERneg.to.ERpos\_collapsed |
| Phenotype | NoPhenotypeAvailable |
| Upregulated in class | na\_neg |
| GeneSet | AGUIRRE\_PANCREAS\_CHR17 |
| Enrichment Score (ES) | -0.50637454 |
| Normalized Enrichment Score (NES) | -2.2228296 |
| Nominal p-value | 0.0 |
| FDR q-value | 5.57833E-4 |
| FWER p-Value | 0.0090 |
Table: GSEA Results Summary

  

Fig 1: Enrichment plot: AGUIRRE\_PANCREAS\_CHR17      
 Profile of the Running ES Score & Positions of GeneSet Members on the Rank Ordered List

  

| PROBE | GENE SYMBOL | GENE\_TITLE | RANK IN GENE LIST | RANK METRIC SCORE | RUNNING ES | CORE ENRICHMENT || 1 | CDC27 |  |  | 523 | 4.294 | -0.0035 | No |
| 2 | FLJ10120 |  |  | 2133 | 2.430 | -0.1045 | No |
| 3 | KIAA0195 |  |  | 2461 | 2.219 | -0.1106 | No |
| 4 | AOC2 |  |  | 2776 | 2.053 | -0.1171 | No |
| 5 | KPNB1 |  |  | 3125 | 1.876 | -0.1276 | No |
| 6 | PSME3 |  |  | 3489 | 1.714 | -0.1406 | No |
| 7 | SPAG9 |  |  | 3926 | 1.544 | -0.1606 | No |
| 8 | NKIRAS2 |  |  | 4766 | 1.231 | -0.2136 | No |
| 9 | PCTP |  |  | 4843 | 1.207 | -0.2092 | No |
| 10 | PSMC3IP |  |  | 5109 | 1.113 | -0.2199 | No |
| 11 | ANKRD40 |  |  | 5202 | 1.080 | -0.2178 | No |
| 12 | EZH1 |  |  | 5481 | 0.979 | -0.2305 | No |
| 13 | STAT5B |  |  | 5880 | 0.852 | -0.2534 | No |
| 14 | MLX |  |  | 5973 | 0.823 | -0.2535 | No |
| 15 | WNT3 |  |  | 6241 | 0.732 | -0.2675 | No |
| 16 | RPL27 |  |  | 6815 | 0.546 | -0.3062 | No |
| 17 | GOSR2 |  |  | 6978 | 0.496 | -0.3142 | No |
| 18 | MPP3 |  |  | 7045 | 0.471 | -0.3153 | No |
| 19 | GPATC8 |  |  | 7264 | 0.398 | -0.3284 | No |
| 20 | UTP18 |  |  | 7481 | 0.316 | -0.3420 | No |
| 21 | NMT1 |  |  | 7789 | 0.193 | -0.3636 | No |
| 22 | NME1 |  |  | 7807 | 0.187 | -0.3633 | No |
| 23 | ARL17P1 |  |  | 7887 | 0.151 | -0.3680 | No |
| 24 | ATP5G1 |  |  | 8013 | 0.100 | -0.3766 | No |
| 25 | LRRC59 |  |  | 8052 | 0.087 | -0.3787 | No |
| 26 | NME2 /// NME1-NME2 |  |  | 8311 | -0.002 | -0.3982 | No |
| 27 | SMARCE1 |  |  | 9023 | -0.322 | -0.4491 | No |
| 28 | LRRC37A /// FLJ10120 |  |  | 9099 | -0.362 | -0.4518 | No |
| 29 | HLF |  |  | 9113 | -0.367 | -0.4497 | No |
| 30 | DUSP3 |  |  | 9123 | -0.371 | -0.4472 | No |
| 31 | CROP |  |  | 9177 | -0.392 | -0.4479 | No |
| 32 | AARSD1 |  |  | 9313 | -0.460 | -0.4543 | No |
| 33 | CBX1 |  |  | 9417 | -0.522 | -0.4577 | No |
| 34 | ATP6V0A1 |  |  | 9795 | -0.721 | -0.4801 | No |
| 35 | PHB |  |  | 10007 | -0.852 | -0.4889 | No |
| 36 | PDK2 |  |  | 10207 | -0.962 | -0.4958 | No |
| 37 | ACLY |  |  | 10348 | -1.060 | -0.4975 | Yes |
| 38 | COX11 /// COX11P |  |  | 10441 | -1.113 | -0.4951 | Yes |
| 39 | TMUB2 |  |  | 10577 | -1.224 | -0.4950 | Yes |
| 40 | CASKIN2 |  |  | 10617 | -1.258 | -0.4874 | Yes |
| 41 | GRN |  |  | 10630 | -1.268 | -0.4777 | Yes |
| 42 | FAM117A |  |  | 10781 | -1.375 | -0.4775 | Yes |
| 43 | COX11 |  |  | 10792 | -1.383 | -0.4667 | Yes |
| 44 | HEXIM1 |  |  | 10897 | -1.472 | -0.4622 | Yes |
| 45 | LOC388397 |  |  | 11295 | -1.819 | -0.4769 | Yes |
| 46 | SLC35B1 |  |  | 11337 | -1.865 | -0.4643 | Yes |
| 47 | RSAD1 |  |  | 11397 | -1.930 | -0.4526 | Yes |
| 48 | KRT10 |  |  | 11580 | -2.108 | -0.4487 | Yes |
| 49 | NPEPPS /// LOC649270 |  |  | 11765 | -2.283 | -0.4434 | Yes |
| 50 | SNF8 |  |  | 11795 | -2.321 | -0.4261 | Yes |
| 51 | MPP2 |  |  | 11798 | -2.325 | -0.4068 | Yes |
| 52 | EPN3 |  |  | 11835 | -2.355 | -0.3898 | Yes |
| 53 | MYST2 |  |  | 11938 | -2.484 | -0.3766 | Yes |
| 54 | SPOP |  |  | 11984 | -2.544 | -0.3587 | Yes |
| 55 | SNX11 |  |  | 12162 | -2.795 | -0.3486 | Yes |
| 56 | UBE2Z |  |  | 12234 | -2.894 | -0.3297 | Yes |
| 57 | ITGA3 |  |  | 12256 | -2.918 | -0.3069 | Yes |
| 58 | FLJ20920 |  |  | 12373 | -3.057 | -0.2900 | Yes |
| 59 | SPATA20 |  |  | 12395 | -3.088 | -0.2657 | Yes |
| 60 | ZNF652 |  |  | 12454 | -3.182 | -0.2434 | Yes |
| 61 | BECN1 |  |  | 12543 | -3.326 | -0.2221 | Yes |
| 62 | XYLT2 |  |  | 12564 | -3.363 | -0.1954 | Yes |
| 63 | PNPO |  |  | 12608 | -3.447 | -0.1698 | Yes |
| 64 | RAB5C |  |  | 12658 | -3.537 | -0.1438 | Yes |
| 65 | NPEPPS |  |  | 12675 | -3.568 | -0.1151 | Yes |
| 66 | NSF |  |  | 12697 | -3.607 | -0.0865 | Yes |
| 67 | G6PC3 |  |  | 12710 | -3.639 | -0.0569 | Yes |
| 68 | NBR1 /// LOC653347 |  |  | 12721 | -3.658 | -0.0270 | Yes |
| 69 | LOC162427 |  |  | 12821 | -3.934 | -0.0014 | Yes |
| 70 | CCDC56 |  |  | 13052 | -4.655 | 0.0202 | Yes |
Table: GSEA details [plain text format]

  

Fig 2: AGUIRRE\_PANCREAS\_CHR17: Random ES distribution      
 Gene set null distribution of ES for **AGUIRRE\_PANCREAS\_CHR17**
